# *Mycobacterium tuberculosis* in patients with hematologic malignancies in a low-risk setting

**DOI:** 10.5588/ijtldopen.25.0155

**Published:** 2025-06-13

**Authors:** L. Mezzadri, A. Tebaldi, V. Ravasio, D. Ripamonti

**Affiliations:** ^1^ School of Medicine and Surgery, University of Milan-Bicocca, Monza, Italy;; ^2^ Infectious Diseases Unit, ASST Papa Giovanni XXIII, Bergamo, Italy.

**Keywords:** tuberculosis, immunosuppression, TB infection, LTBI

*Mycobacterium tuberculosis* infects nearly a quarter of the world's population and active TB causes high morbidity and mortality.^1^ Following exposure, approximately 30% of individuals develop latent TB infection (LTBI), and without treatment around 10% of immunocompetent individuals will progress to active disease, half within the first two years.^2^ This risk is substantially higher in immunocompromised populations, including those with HIV, end-stage renal disease, solid organ transplants, cancer, or those receiving chemotherapy, high-dose steroids, or biological agents.^3^ Given the increased risk of progression, LTBI screening with tuberculin skin test (TST), or interferon-gamma release assays (IGRAs) is recommended for certain individuals, such as recent contacts of untreated TB cases, immunocompromised individuals, and those living in TB-prevalent areas at moderate risk of reactivation.^2^ In Italy, a low-incidence region, LTBI prevalence in patients with hematologic malignancies and hematopoietic stem cell transplantation (HSCT) is estimated at 7–8%.^4,5^ However, the risk of TB reactivation in these patients is approximately 2–40 times higher than in the general population.^4^ Recent data confirm the feasibility and good tolerability of TB prophylaxis in hematologic settings,^6^ However, the interpretation of IGRA testing may be difficult in an immunocompromised population due to the higher proportion of indeterminate results compared to immunocompetent persons.^7^ Additionally, uncertainties persist regarding screening and preventive treatment, even when evidence of LTBI is documented. For example, current guidelines on LTBI screening and treatment in patients with hematological malignancies suggest an individualized approach, targeting only high-risk patients, such as those from high-incidence TB areas, individuals exposed to contagious TB, patients with pleuro-parenchymal imaging suggestive of previous TB, and those receiving ruxolitinib with substantial epidemiological risk factors.^8^ Decisions regarding screening and initiating TB prophylaxis should also consider the patient’s prognosis, age, and the benefit-risk balance.^8^ A recent survey highlights the heterogeneity in LTBI screening across Italian hematology centers. Most centers screen only at-risk patients, such as those from high-endemic regions, with previous TB exposure, or chest imaging abnormalities, while some routinely screen patients undergoing moderate to intensive anti-leukemic treatments, regardless of clinical history.^9^

In this heterogenous scenario and without a clear-cut universal screening recommendation, a high index of suspicion for active TB should be maintained in patients with hematologic malignancies presenting with persistent fever unresponsive to broad-spectrum antimicrobials. Here we report on two cases of fatal TB in patients with hematologic malignancies (HMs), which highlight the importance of an aggressive diagnostic approach, even in a low-risk setting, within this vulnerable population.

*Case 1:* In 2022, a Caucasian 56-year-old man, after being diagnosed with acute myeloid leukemia, developed a persistent fever one month after starting chemotherapy. Born in Italy, he had no other comorbidities, and no history of TB or travel to TB-endemic regions. A single IGRA test (QuantiFERON-TB Gold In-Tube, QFT-GIT) was indeterminate. His chemotherapy included daunorubicin, cytosine arabinoside and gemtuzumab. One month later, bone marrow aspiration confirmed the remission of leukemia. Due to the development of lung infiltrates, the patient was started on vancomycin and meropenem, and following the worsening pulmonary disease switched to piperacillin/tazobactam, amikacin, ceftaroline and amphotericin B. The patient developed hemophagocytic lymphohistiocytosis (HLH), requiring steroids and immunoglobulin therapy. TB diagnosis was finally confirmed using GeneXpert® MTB/RIF Ultra on bronchoalveolar lavage (BAL) performed the day before his death, 46 days after hospital admission.

*Case 2:* In 2023, a Caucasian 75-year-old man, after being diagnosed with tricholeukemia, developed persistent fever one month after starting chemotherapy. He had a history of ulcerative colitis successfully treated with mesalazine alone. He was born in Italy, he also reported no history of TB or previous travelling to TB endemic regions. After treatment with rituximab and a 5-day cycle of cladribine, he developed HLH, requiring steroids plus immunoglobulins. Due to the occurrence of fever and pulmonary disease (multiple opacities, mediastinal lymph adenopathies and bilateral pleural effusions), he was empirically treated with meropenem, ceftaroline and amphotericin B and transferred to the intensive care unit (ICU). TB diagnosis was confirmed by blood culture performed 14 days prior. He was started on a TB regimen with five-drugs (isoniazid, ethambutol, amikacin, levofloxacin and pyrazinamide), avoiding rifampicin due to drug interactions. He died one week later, 54 days after hospital admission.

Both cases highlight the challenges of diagnosing active TB in immunocompromised patients. A summary of the clinical characteristics and outcomes of these two patients is shown in the Table. Persistent fever in patients with HMs, without microbiological diagnosis and unresponsive to empirical antibacterial and antifungal regimens, is not infrequent and represents a clinical dilemma. In the context of immunosuppression, viral reactivation (such as for CMV and EBV) or fungal diseases (such as aspergillosis, cryptococcosis and *Pneumocystis jirovecii* disease) are expected events, cancer-related fever is an option, whereas drug-induced fever is largely based on exclusion criteria, and it is confirmed by drug discontinuation. Clinicians should consider the risk of TB reactivation even in low-risk areas, and in patients without known or past exposure to TB, especially for those on a high dose of steroids. This challenge persists as typical radiological findings can be subtle or absent in immunosuppressed patients,^5^ whereas IGRA testing may yield inconclusive results for LTBI diagnosis in this setting.^10^ A recent meta-analysis investigating new generation IGRAs suggests that the diagnostic performance of QuantiFERON-TB Gold Plus (QFT-Plus) is similar to that of QFT-GIT and T-SPOT.TB TB assay.^11^ An IGRA is preferred for testing persons who have received Bacillus Calmette-Guérin for cancer therapy or previous vaccination.^12^

**Table.** Clinical characteristics and outcomes of two patients with active TB and hematologic malignancies.

	Case 1	Case 2
Age (years), Gender	56, male	75, male
Country	Northern Italy	Northern Italy
Hematologic malignancy	Acute Myeloid Leukemia	Tricholeukemia
Comorbidities	None	Ulcerative Colitis
Chemotherapy	Daunorubicin, Cytarabine, Gemtuzumab	Rituximab, Cladribine
Complications	Hemophagocytic Lymphohistiocytosis, respiratory failure	Hemophagocytic Lymphohistiocytosis, respiratory failure
Pulmonary findings	Lung infiltrates	Multiple opacities, mediastinal lymphadenopathy, bilateral pleural effusions
TB risk factors	None identified	None identified
IGRA test	Indeterminate	Not performed
Time from admission to TB diagnosis	46 days (post-mortem diagnosis)	40 days (14 days before death)
Diagnostic method	PCR on BAL fluid	Blood culture
TB treatment	None (post-mortem diagnosis)	isoniazid, ethambutol, amikacin, pyrazinamide
Outcome, (days since hospital admission)	Death (46)	Death (54)

BAL = bronchoalveolar lavage; IGRA = interferon-gamma release assay; PCR = polymerase chain reaction**.**

Starting a patient on empiric TB treatment is a complex decision as it requires a multidrug regimen, with a high potential for drug-to-drug interactions (mostly due to rifampin) and for the risk of drug-associated liver toxicity. Data from the literature confirms that most bloodstream infections in HMs are caused by bacteria,^13^ and even in high-endemic regions, TB reactivation remains infrequent.^13^ Although extrapulmonary TB manifestations are possible in up to one third of patients with HMs, pulmonary involvement is a frequent event, and the presence of mediastinal lymphadenopathy, pleural effusions and fibrocalcified lesions should alert clinicians.^14^ An early and aggressive diagnostic approach should be the standard when pulmonary involvement is present, and although BAL investigation may be complicated in some clinical conditions, its diagnostic yield is suboptimal in immunocompromised patients and complications are frequent.^15^ Prophylactic platelet transfusions (when platelet count is below 50,000) are typically administered prior to an invasive procedure to patients at significant risk for bleeding, although largely based on expert opinion. Other authors, suggest that the risk for bleeding following this procedure is minimal, even at lower platelet count.^16^ However, less invasive diagnostic samples such as sputum, or gastric aspirate may be useful in thrombocytopenic patients as an alternative to bronchoscopy. As for diagnostic approaches, a recent meta-analysis on metagenomic next generation sequencing (mNGS) technology showed a high sensitivity and speed in diagnosing *Mycobacterium tuberculosis* and mixed infections.^17^

Our two cases confirm that, in patients with hematologic malignancies, a high degree of suspicion is required for TB. An early and aggressive diagnostic work out is crucial to optimize treatment for such infrequent, but potentially curable, infectious complications.
